# Novel PVA-Based Microspheres Co-Loaded with Photothermal Transforming Agent and Chemotherapeutic for Colorectal Cancer Treatment

**DOI:** 10.3390/pharmaceutics13070984

**Published:** 2021-06-29

**Authors:** Yao Zhang, Zirui He, Fan Yang, Changqing Ye, Xia Xu, Shige Wang, Ling Zhang, Duowu Zou

**Affiliations:** 1 Department of Gastroenterology, Ruijin Hospital Affiliated to Shanghai Jiao Tong University School of Medicine, No.197, Rui Jin Er Road, Shanghai 200025, China; zyrjxh97@sjtu.edu.cn; 2 Department of General Surgery, Ruijin Hospital Affiliated to Shanghai Jiao Tong University School of Medicine, No.197, Rui Jin Er Road, Shanghai 200025, China; hzr11770@rjh.edu.com; 3 College of Science, University of Shanghai for Science and Technology, No. 334 Jungong Road, Shanghai 200093, China; yfbetter@163.com (F.Y.); yegreenleaf@163.com (C.Y.); xia210882812@163.com (X.X.); sgwang@usst.edu.cn (S.W.)

**Keywords:** electrospinning, nanospheres, chemotherapy, photothermal tumor therapy, colorectal cancer

## Abstract

Background: We previously designed an electrospinning chitosan (CS) nanofiber-based carrier, using polyvinyl alcohol (PVA) as an adjuvant to deliver doxorubicin (DOX) and MoS_2_ nanosheets for postoperative tumor re-occurrence inhibition. However, owing to that the nanofibrous mat is un-injectable, this composite nanofiber is far from being clinically applicable. Materials and Methods: Via modulating the electrospray parameters, polyvinyl alcohol (PVA) beads string doped with DOX and MoS_2_ (PVA/MoS_2_/DOX microspheres) were prepared, which were further crosslinked with glutaraldehyde to obtain the water-stability. Results: Under the 808-nm laser irradiation, MoS_2_ nanosheets rendered the prepared PVA/MoS_2_/DOX microspheres an excellent light-to-heat conversion performance with η of 23.2%. Besides, the heat generated by near-infrared laser irradiation can improve the effect of chemotherapy by promoting the release rate of DOX. HT29 cell and tumor-bearing nude mice were used to systematically study the combined tumor treatment efficiency of composite nanospheres. Conclusion: PVA/MoS_2_/DOX nanospheres have excellent photothermal effect and chemotherapy effect, which can completely suppress the tumor recurrence. Therefore, the PVA/MoS_2_/DOX nanospheres are anticipated to find potential applications in the treatment of local colorectal cancer.

## 1. Introduction

Colorectal cancer (CRC) annually kills about 700,000 people, making it the world’s fourth most deadly cancer beyond stomach, liver, and lung cancer [1]. Owing to the advances in early detection and treatment for CRC, approximately 64.9% of CRC survivors could live five years after their diagnosis [2,3]. Although the encouraging long-term therapy outcome of CRC, oncologists are facing new challenges for current treatments. Surgery treatment makes some patients live with a permanent ostomy, resulting in a lower physical quality of life [4]. Since most anticancer drugs affect both normal cells and cancer tissue, chemotherapy is always accompanied by side effects [5,6,7]. Moreover, about 50% of the surgically treated patients will experience a recurrence within the first three years after that [8]. In light of the above drawbacks of the conventional therapies, seeking more effective, specific, and minimally invasive treatment methods of CRC are necessary.

As a broad-spectrum anti-tumor anthracycline, doxorubicin (DOX) is being widely used to treat various cancers [9,10]. DOX cannot be used for intravenous administration directly because of its hydrophobicity. Although DOX can be changed into soluble drugs or dissolved with solutions containing surfactants, both of these methods can reduce the sensitivity and cause other side effects [11]. Microsphere as a new drug carrier has great potential in local treatment of tumors. Photothermal therapy (PTT) as a novel treatment method with fewer side-effects has the advantage over radiotherapy and chemotherapy [12,13]. During PTT, photothermal agents with high efficiency of photothermal conversion are injected into the living body and gathered in the tumor tissue. Then, under the irradiation of near-infrared (NIR) light, the light energy is converted into heat energy to kill the tumor cells via hyperthermia [14]. At present, the main research materials for PTT can be divided into four categories: noble metal nanomaterials (like Au and Ag), carbon nanomaterials (like graphene and carbon nanotubes), metallic compounds (like CuS and MoS_2_), and organic dyes (like ICG) [15,16,17]. Among them, MoS_2_ has obtained exciting results in the PTT of the tumor [18,19].

**Scheme 1:**
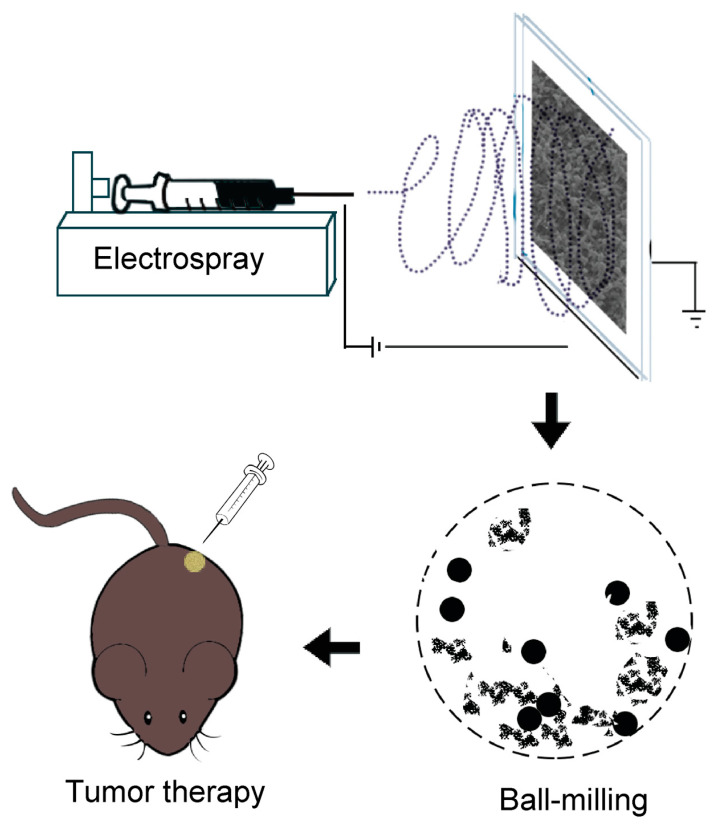
Schematic illustration of the electrospray, ball-mailing, and tumor therapy.

Electrospinning has found tremendous applications in the high-yield production of nano-/micro-spheres and fibrous mats [20,21]. A large number of studies have proved the safety and effectiveness of nano-/micro-spheres and fibrous mats in the treatment of a variety of tumors [22,23]. In a previous study, we designed an electrospinning polyvinyl alcohol (PVA)/chitosan (CS) nanofiber-based composite carrier to deliver DOX and MoS_2_ nanosheets for postoperative tumor treatment [24]. However, owing to the fact that the nanofibrous mat is un-injectable, this composite nanofiber is far from being clinically applicable. In this study, we are committed to using microspheres as a novel drug-delivery system, to co-load chemotherapy drugs and photothermal agents to achieve the effect of combination therapy. Via modulating the electrospinning parameters, PVA beads doped with DOX and MoS_2_ (PVA/MoS_2_/DOX microspheres) were prepared, which were further crosslinked with glutaraldehyde to obtain the water-stability. Finally, we developed a new experimental method using the ball-mailing which can grind the water-stable beads into microspheres. In order to study the in vivo tumor therapy efficiency, the obtained PVA/MoS_2_/DOX microspheres were locally injected into the colorectal tumor sites of the tumor-bearing Balb/c nude mice, and an NIR laser with a wavelength of 808 nm was used to irradiate the PVA/MoS_2_/DOX microspheres in the tumor (Scheme 1). In this formulation, the release of DOX is sustainable and the MoS_2_ nanosheets are restricted in the microspheres, therefore, a highly efficient and safe dual model tumor PTT and chemotherapy was realized. It is anticipated that this work will shed light on the promotion of the biomedical application of the electrospinning materials.

## 2. Materials and Methods

### 2.1. Electrospinning

The commercially available PVA with low viscosity and high viscosity (Aladdin reagent, Shanghai) were respectively dissolved in acetic acid (70 wt.% in water) and magnetically stirred for 2 h at 80 °C to form two solutions with the concentration of 30 wt.%. The viscosity of high viscosity PVA is 50.0–65.0 mPa∙s and the viscosity of low viscosity PVA is 5.0–7.0 mPa∙s. The two solutions were mixed at a volume ratio of 43:7 (low viscosity: high viscosity) for electrospinning. After that, MoS_2_ nanosheets that were prepared according to our previous research [18] and DOX (Beijing Huafeng Pharmaceutical Co., Ltd., Beijing, China) were mixed with the PVA solution for electrospinning under ambient conditions to prepare PVA/MoS_2_ and PVA/MoS_2_/DOX microspheres. DOX and MoS_2_ nanosheets concentrations were set as 10 and 6 mg/mL, respectively. PVA/MoS_2_/DOX PVA/MoS_2_ and PVA microspheres were prepared via a home-made electrospinning machine. The feeding rate of the solution was 0.5 mL/h, the collecting distance was 15 cm, and the applied voltage was set at 20 kV. After the electrospinning, the microsphere mats were removed from the collector and put in a vacuum oven for drying. All water used in this study with the resistivity >18.2 MΩ·cm was distilled with a Millipore water purification system (Milli-Q Plus 185).

### 2.2. Crosslinking

The formed PVA/MoS_2_/DOX, PVA/MoS_2_ and PVA mats were crosslinked with glutaraldehyde to form hydrophobic mats. The cross-linking solution was composed of 0.40 mL of glutaraldehyde, 0.03 mL of concentrated hydrochloric acid, and 15 mL of acetone. For crosslinking, the microspheres mat was soaked in the glutaraldehyde/concentrated hydrochloric/acetone solution for 0.5 h and washed with phosphate-buffered saline (PBS) three times. The microspheres mat was finally dried for 12 h in a vacuum oven and subjected to a heat treatment at 40 °C. Scanning electron microscope (SEM, JEOL JSM-5600LV, Japan) was used to obtain the surface morphology pictures of the prepared microsphere mats. Then, 50 individual microspheres of each sample were chosen to calculate the diameter of microfibers using Image J 1.40 G software. Chemicals in this Section 2.2 were bought from Sinopharm Chemical Reagent Co., Ltd., Shanghai, China.

### 2.3. Ball-Mailing Process

The PVA, PVA/MoS_2,_ and PVA/MoS_2_/DOX microspheres were produced by ball-mailing the crosslinked microsphere mats obtained in the previous steps for 2 h, using a planetary ball-mailing machine (UBE-V0.2L, Changsha Deco Equipment Co., Ltd., Changsha, China) in a stainless steel vessel which rotated at a speed of 180 rpm. The surface morphology of microspheres was characterized by SEM.

### 2.4. In Vitro Photothermal Performance of PVA-Based Microspheres

To explore the photothermal performance of PVA (set as control) and PVA/MoS_2_ microspheres, they were added into the cell culture hole of a 24-well plate with the concentration of 2.5 mg/mL and then continuously irradiated with an NIR laser (DL-808-070-T, SFOLT Co. Ltd., Shanghai, China) for 5 min with a power density of 1.0, 0.5, or 0.3 W/cm^2^. The temperature rise (ΔT) of the irradiated microspheres was recorded with a FLIR™ E60 infrared camera (FLIR, San Francisco, CA, USA). In order to test the photothermal stability, the PVA/MoS_2_ microspheres in water (2.5 mg/mL) were irradiated for 5 min (power density: 1 W/cm^2^) and the laser was then turned off to naturally cool the microspheres to room temperature. This laser on/off irradiation was repeated for five cycles. The photothermal conversion efficiency (η) under 808 nm NIR laser irradiation was calculated using Equation (1) in the Supporting Information [25].

### 2.5. Drug Loading and Release

The drug loading performance of the PVA/MoS_2_/DOX microspheres was evaluated using a Shimadzu UV-3600 UV-vis-NIR spectrometer (Shimadzu, Kyoto, Japan). The crosslinking solution’s absorbance at 490 nm was read and the concentration of free DOX was then calculated as per the pre-fixed standard curve. The DOX loading efficiency of PVA/MoS_2_/DOX microspheres was calculated based on its concentration in a crosslinking solution. For the drug release study, 5 mg PVA/MoS_2_/DOX microspheres were placed in a dialysis tube that was filled with 5 mL of sodium acetate–acetic acid buffer solution (pH = 5.4, to simulate the tumor circumstance) or 5 mL PBS (pH = 7.4, to simulate the physiological circumstance). These glass vials were incubated at 37 °C and stirred (stirring rate: 90 rpm). At the predetermined time points, 1.5 mL of the released solution was taken from each vial and a 1.5 mL fresh buffer solution was added. A UV-Vis spectrophotometer (Shimadzu, Kyoto, Japan) was used to read the absorbance of the released solution (wavelength: 490 nm). The DOX concentration was recorded as per the pre-fixed standard curves (at pH = 7.4 and 5.4).

### 2.6. In Vitro Biocompatibility and Hemocompatibility

To evaluate the in vitro biocompatibility, we used mouse fibroblast and human colorectal carcinoma (HT29) cell lines. All the cells were purchased from the Chinese Academy of Sciences and cultured in a standard humidified incubator. The used medium is DMEM containing streptomycin (100 μg/mL), penicillin (100 units/mL), and FBS (10%, *v*/*v*). Before the experiment, microspheres were sterilized with 75% ethanol for 2 h. Then, the PVA or PVA/MoS_2_ microspheres were put in the transwell (2.5 mg/mL) and added into the 24-well cell culture well which was seeded with cells (5 × 10^4^ cells in each well). Cells cultured with pure DMEM were set as another control. After being cultured for 24 h, the survival rate of cells in each group was measured using the cell counting kit-8 (CCK-8) and Dead/Live staining. The images were read with a Leica DM IL LED (Atlanta, GA, USA) inverted phase-contrast microscope.

The in vitro hemocompatibility assay of PVA and PVA/MoS_2_ microspheres was performed using mice red blood cells (mRBC) as a model. To this end, PBS (4 mL) and distilled water (4 mL) served as positive control and negative control, respectively, which was mixed with mRBCs (1 mL, in PBS). In experimental groups, 12.5 mg PVA or PVA/MoS_2_ microspheres dispersed in 4 mL of PBS that were mixed with 1 mL mRBCs. These specimens were incubated (37 °C, 2 h) and centrifuged (10,000 rpm, 2 min) to get the supernatant for absorbance reading at 541 nm with a Shimadzu UV-3600 UV-vis-NIR spectrometer (Shimadzu, Kyoto, Japan). The absorbance of the supernatant from PBS, DI water and microspheres treated blood cells were recorded as *D_nc_*, *D_pc_*, and *D_t_* respectively. The calculation formula of hemolysis percentage (*HP*) is as follows [26]:

$$HP\left(\%\right)=\frac{\left(Dt-Dnc\right)}{\left(Dpc-Dnc\right)}\times 100\%$$

### 2.7. In Vivo Hemocompatibility

All animal experiments were approved by the Institutional Animal Care and Use Committee of Shanghai Jiao Tong University (Y20190358) and performed following the National Institutes of Health guidelines for the use of experimental animals. The in-vivo hemocompatibility of PVA/MoS_2_ microspheres was evaluated by serum biochemical and routine blood tests and compared with healthy mice (control). Sysmex-XS-800i automatic blood analyzer (Atlanta, GA, USA) was used for blood routine parameter detection and DxC 800 automatic biochemical analyzer (Atlanta, Georgia, USA) was used for serum biochemical detection. To test the blood parameters, 5 mg of PVA/MoS_2_ microsphere mats were subcutaneously implanted in the KM mice. Blood collection time was 7 and 28 days, respectively, after microspheres’ injection. Mice were anesthetized with a cardiac puncture to collect blood. The studied blood parameters are listed in the Supplementary Materials. Blood taken from Healthy KM mice served as the control.

### 2.8. Biodistribution of Mo Ions and Histocompatibility

KM mice were used as models to analyze the biological distribution of Mo ions. Briefly, KM mice, subcutaneously implanted with PVA/MoS_2_ microspheres (5 mg), were euthanized 7 or 28 days after feeding. Aqua regia was used to digest the main organs to form a homogenous solution. The Agilent 700 series IPVA-OES was used to quantify the Mo ion concentration in the above solutions. The standard H&E staining method was used to study the in vivo histocompatibility of PVA/MoS_2_ microspheres. Then, 5 mg of PVA (control group) and PVA/MoS_2_ microspheres were implanted subcutaneously in KM mice. The staining was carried out at day 7 and day 28, respectively, after the microspheres’ implantation. The images were read with a Leica DM IL LED (Atlanta, GA, USA) inverted phase-contrast microscope. The treated KM mice were also weighed during the feeding. The studied main organs in this section include heart, liver, spleen, lung, and kidney.

### 2.9. In Vitro Tumor Therapeutic Efficiency

A total number of 5 × 10^4^ HT29 cells were seeded in the wells of a 24-well plate and routinely cultured for 24 h. Then, with the blank culture solution as the control, 5 mg of sterilized PVA, PVA/DOX, PVA/MoS_2_, or PVA/MoS_2_/DOX microspheres was added into the wells of a 24-well plate (containing 2.5 mL DMEM per well). The cells were then irradiated with NIR laser (808 nm, 1.0 W/cm^2^, 5 min), the metabolic activity of HT29 cells was measured by Dead/Live kit and CCK-8 kit, using an MK3 micro-plate reader (Thermo Fisher, Waltham, MA, USA) and Olympus BX43 fluorescence microscope, respectively.

### 2.10. Postoperative Tumor Recurrence Inhibition Study

To construct the tumor model, HT29 cells (10^7^ cells in 0.1 mL serum free DMEM) were subcutaneously injected into the back of Balb/c nude mice. These mice were randomly divided into 4 groups. After 3 weeks of feeding, the tumor nodules (about 1 cm in diameter) were surgically removed. The evaluation of the tumor recurrence suppression effect was performed as follows: 5 mg PVA (control), PVA/DOX, PVA/MoS_2_, or PVA/MoS_2_/DOX microspheres was implanted in the scar after tumor removal and the scar was glued with a bio-glue (*n* = 6). The scar was irradiated with a NIR laser for 5 min (1 W/cm^2^). A FLIR™ E60 camera was used to record the thermal image and the temperature of the laser-illuminated mice. The tumor recurrence was evaluated by measuring the tumor volume and the appearance of the tumor was also recorded to evaluate the effect of postoperative treatment of the tumor.

### 2.11. Statistical Analysis

The significance of the experimental data in this research was studied using the one-way ANOVA statistical analysis method. A value of 0.05 was fixed as the threshold, and the figures were indicated with (*) if their probability were less than 0.05 (*p* < 0.05), (**) if *p* < 0.01, or (***) if *p* < 0.001. Unless specified, the sample size is 3 (*n* = 3).

## 3. Results and Discussions

### 3.1. Microsphere Mats Preparation and Characterization

**Figure 1:**
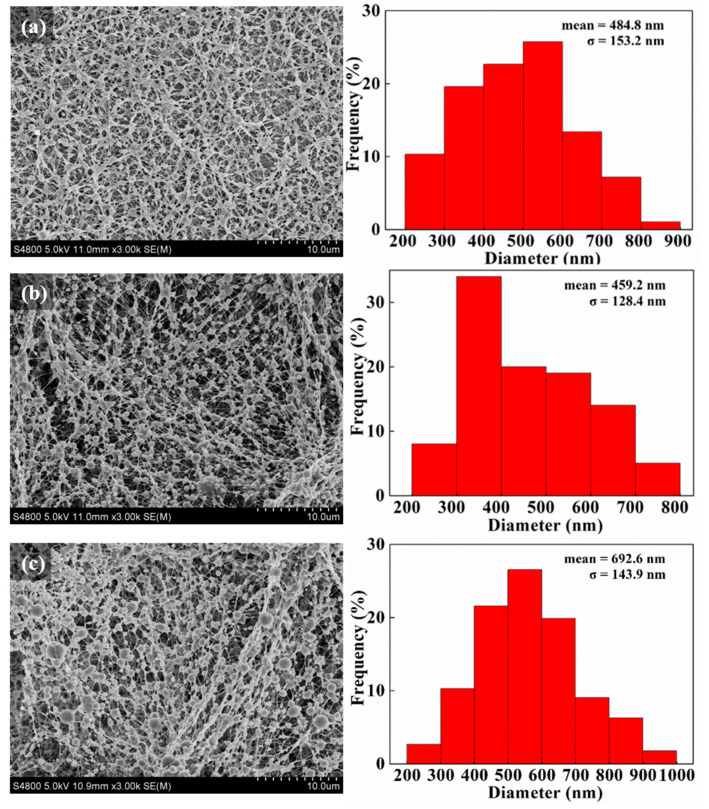
SEM pictures and diameter distribution histograms of PVA (**a**), PVA/MoS_2_ (**b**), and PVA/MoS_2_/DOX (**c**) microspheres.

**Figure 2:**
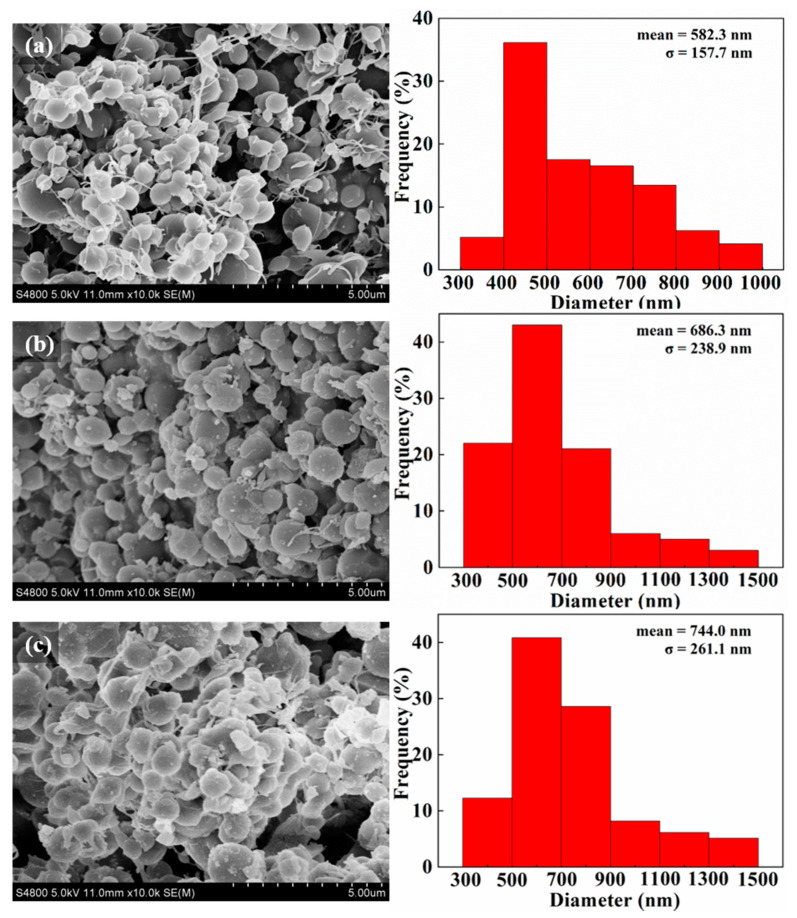
SEM pictures and diameter distribution histograms of crosslinked PVA (**a**), PVA/MoS_2_ (**b**), and PVA/MoS_2_/DOX (**c**) microspheres.

According to our previous research, we firstly prepared MoS_2_ nanosheets with a diameter of 20 nm [27] and used the bend electrospinning method to prepare PVA-based composite microspheres from their solutions. The proportion of high-viscosity and low-viscosity PVA determines whether the resultant product PVA can form a bead-like structure during the electrospinning. As found, at the volume ratio of 43:7, the obtained product shows a “beaded structure”, which is beneficial to the formation of PVA/MoS_2_/DOX microspheres after the ball-mailing. For comparison, PVA and PVA/MoS_2_ microspheres were also prepared from PVA and PVA/MoS_2_ solutions, respectively. The potential formation mechanism of PVA-based microspheres can be explained as the break of the balance between the solution surface tension and the repulsive force among the charged polymer chains. The PVA solution was charged during the electrospinning process and these charged polymer chains repulsed mutually. When the electric field strength is higher than the threshold value, the polymer solution was ejected from the needle of the syringe and traveled to the collector, during which the water was evaporated and the PVA chains was twisted in spheres. The microspheres were further crosslinked with glutaraldehyde to obtain the water-stability. After the preparation process, we used SEM to characterize the morphologies of the formed microspheres. As shown in Figure 1a, the cross-linked PVA formed a bead-like structure with a particle size of 484.8 ± 153.2 nm. After 5 h of ball-mailing, the beads became separated microspheres with a diameter of 582.3 ± 157.7 nm (Figure 2a). The particle size of PVA/MoS_2_ microspheres was 459.2 ± 128.4 nm (Figure 1b). After ball-mailing, the diameter of PVA/MoS_2_ microspheres decreased to 686.3 ± 238.9 nm (Figure 2b). Figure 1c shows that the particle size of the crosslinked PVA/MoS_2_/DOX microspheres was 692.6 ± 143.9 nm. After ball-mailing, the diameter increased to 744.0 ± 261.1 nm (Figure 2c). The diameters of PVA, PVA/MoS_2_, and PVA/MoS_2_/DOX microspheres were increased, probably because the heat and squeezing effects of the ball-mailing process can lead to the swelling of the polymer microspheres.

### 3.2. Photothermal Performance

**Figure 3:**
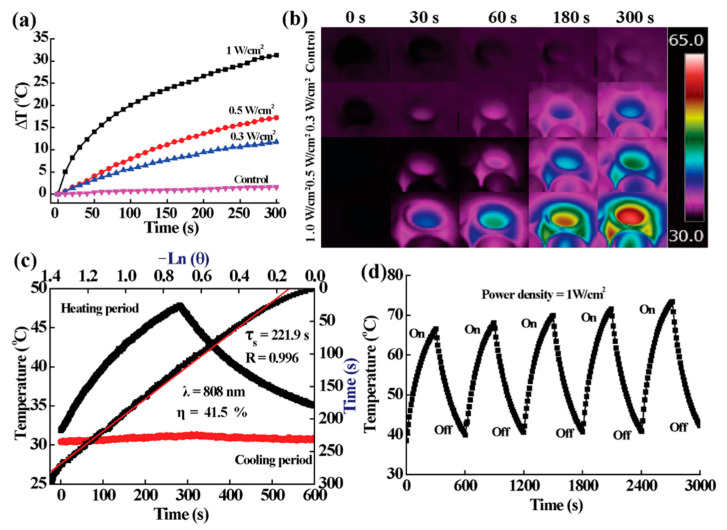
(**a**,**b**) The ΔT curves and thermal pictures of MoS_2_-containing PVA-microspheres after the laser irradiation at the noted power densities. (**c**) The calculation of η and the time constant for heat transfer of MoS_2_-containing PVA-microspheres. (**d**) Temperature curve containing PVA-microspheres during 5 cycles of laser on and off.

MoS_2_ is a member of the transition metal sulfide family and has been proven to have good photothermal conversion efficiency and biocompatibility in previous studies [28,29]. In our previous study, the UV-Vis-NIR spectrum of MoS_2_ proved that MoS_2_ had an obvious light absorption [18,29]. Therefore, we selected 808 and 1064 nm, respectively, as the light source to explore the photothermal performance of PVA/MoS_2_ microspheres. It was confirmed that the photothermal conversion results of PVA/MoS_2_ microspheres are related to the irradiation time and the power density of the NIR laser (Figure 3). Under the 5-min 808-nm laser irradiation (laser power densities: 1.0, 0.5, and 0.3 W/cm^2^), the ΔT were measured as 31.4, 17.5, and 11.2 °C respectively after 5 min of laser irradiation (Figure 3a). An infrared camera (FLIR) was used to record the infrared thermal images of PVA/MoS_2_ microspheres under NIR laser radiation to further visually evaluate the photothermal performance. It was found that the high-temperature region quickly expanded and reached the entire coverage of the cell culture pores, further indicating that PVA/MoS_2_ microspheres had excellent photothermal conversion capabilities in vitro (Figure 3b). Under the irradiation of 808-nm laser, the light-to-heat conversion efficiency of PVA/MoS_2_ microspheres is 41.5% (Figure 3c). The photothermal stability of PVA/MoS_2_ microspheres was then studied, which indicates that the temperature changes of PVA/MoS_2_ microspheres during five laser on/off cycles were consistent under the 808- and 1064-nm laser (Figure 3d). Therefore, PVA/MoS_2_ microspheres have admirable photothermal efficiency and stability, which is expected to facilitate their important application in the PTT cancer treatment.

### 3.3. In Vitro Drug Loading and Release Studies

**Figure 4:**
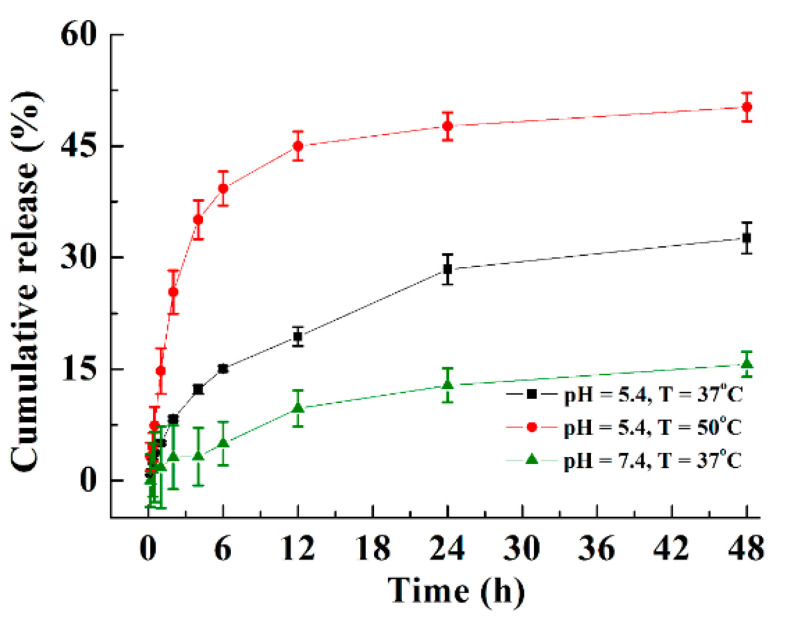
The cumulative release of DOX from DOX and MoS_2_ nanosheets containing PVA microspheres at different pH and temperature values.

As a drug delivery system, PVA/MoS_2_ microspheres can load and control DOX release. Based on the standard curve and the recorded absorbance, the DOX concentration released into the crosslinking solution was 0.91 mg/mL. Therefore, the drug loading amount of PVA/MoS_2_/DOX microspheres was approximately 5.13%. The drug from PVA/MoS_2_/DOX microspheres was characterized with a two-stage profile: a slightly quick release in the first 12 h, and a slow and controlled release in the second stage (the equilibrium-release amounts were about 15.6%, 32.6%, and 50.2% at pH = 7.4, T = 37 °C; pH = 5.4, T = 37 °C; and pH = 5.4, T = 50 °C, respectively, Figure 4). The initial rapid release may be due to the release of the physically adsorbed drug on the microsphere surface, while the controlled drug release suggests the controlled release ability of PVA/MoS_2_/DOX microspheres. Moreover, the above study results also prove that the drug release performance of PVA/MoS_2_/DOX microspheres is sensitive to both temperature and pH, which enables the microspheres to enhance the tumor treatment ability because the generated heat can not only kill tumor cells by high temperature but also increase the release rate of DOX.

### 3.4. In Vitro Cytocompatibility

**Figure 5:**
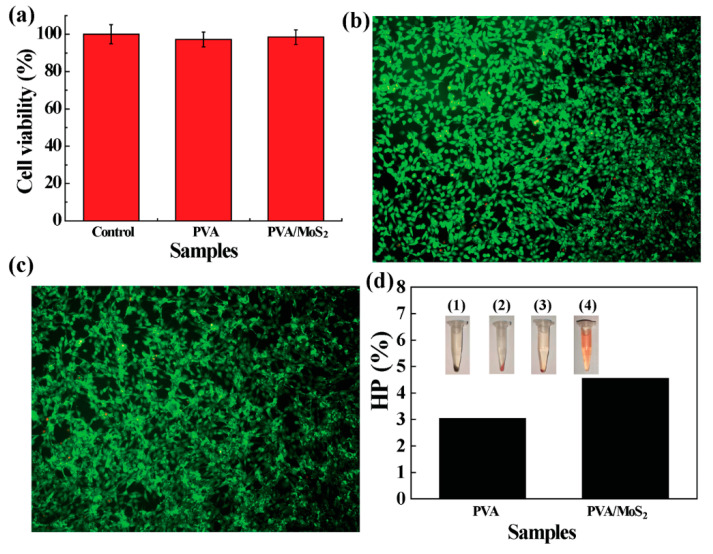
(**a**) The survival rate of HT 29 cells after treatments as noted. (**b**,**c**) Dead/Live staining of cells treated with (**b**) PVA and (**c**) MoS_2_ containing PVA microspheres. (**d**) HP values of mRBCs treated with PVA and MoS_2_ containing PVA microspheres. The inset pictures in panel d are the centrifuged mRBCs that are treated with: (1) saline, (2) PVA, (3) MoS_2_ containing PVA, and (4) water.

The in vitro compatibility of cells was evaluated by measuring the cell survival rate and morphology after culturing cells with PVA and PVA/MoS_2_ microspheres for 24 h. The results show that the survival rate of L929 cells treated with PVA or PVA/MoS_2_ microspheres is higher than 95% (PVA: 99.1%; PVA/MoS_2_: 98.4%), and there was no significant difference compared with untreated L929 cells (incubation time: 24 h, *p* > 0.05, Figure 5a). We further observed the morphology of healthy cells cultured with PVA and PVA/MoS_2_ microspheres using Dead/Live staining. Under the view of an inverted phase-contrast microscope, nearly all cells showed the green color after performing the Dead/Live staining, proving that the non-cytotoxicity of the composite microspheres (Figure 5b,c).

### 3.5. In Vitro Hemocompatibility and In Vivo Biocompatibility

Assessing the in vitro hemocompatibility of PVA and PVA/MoS_2_ microspheres through hemolysis experiments is a prerequisite to ensure their biological safety. PVA, PVA/MoS_2_ microspheres, water (the positive control), and saline (the negative control) were used to treat mRBCs. In physiological saline, the structural integrity of mRBCs remained intact, and the hemolytic rate was set as zero. However, mRBCs can be totally destroyed by water molecules and the hemolytic rate was 100%. The hemolysis rates of PVA and PVA/MoS_2_ microspheres were 1.6 ± 0.3% and 2.3 ± 0.6%, respectively (Figure 5d). As in normal saline, after incubation with PVA and PVA/MoS_2_ microspheres, mRBCs can be easily separated from the solution (inset of Figure 5d), further illustrating that the PVA and PVA/MoS_2_ microspheres did not destroy the structural integrity of mRBCs.

**Figure 6:**
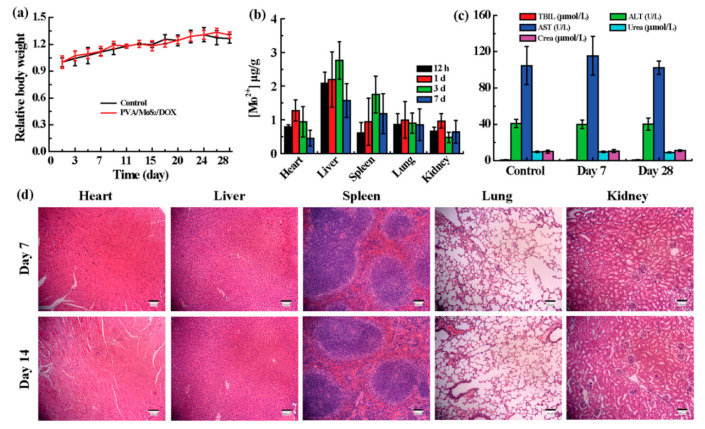
(**a**) The weight gain of healthy and treated mice. (**b**) The Mo distribution in major organs of treated mice. (**c**) The blood biochemistry and (**d**) H&E staining of treated mice. Treated mice are mice that were treated with PVA/MoS_2_/DOX microspheres. AST: aspartate aminotransferase; ALT: alanine aminotransferase; TBIL: total bilirubin. Sale bar = 100 μm (panel (**d**)). In panel (**c**), U/L = unit/L.

In addition to verifying in vitro hemocompatibility, the biocompatibility of microspheres was then researched on healthy KM mice. After subcutaneous implantation of microspheres, KM mice in the experimental group and the control group maintained the same body weight gain trend (Figure 6a). In order to prove that PVA/MoS_2_ microspheres can aggregate in tumor sites and thereby ensuring the efficient tumor treatment and avoiding damage to healthy tissues, the in vivo biological distribution of Mo was studied by ICP. After 7 and 28 days of feeding, we analyzed the concentration of Mo ions in various organs (Figure 6b). It was found that the accumulation of Mo in the kidney, lung, spleen, liver, and heart was limited. For example, the Mo contents in the major organs were lower than 3 μg Mo/g organ in the feeding time points. The biodistribution results indicate that most MoS_2_ nanosheets were embedded in tumors, thereby eliminating their potential threat to normal tissue.

The in vivo compatibility of PVA/MoS_2_ microspheres was also assessed by serum biochemical and routine blood tests on the 7th and 28th day after the subcutaneous implantation of PVA/MoS_2_ microspheres. Compared with control group, the serum biochemical and blood routine parameters of the mice in the experimental group were not significantly changed (Figure 6c and Figures S1 and S2). Consistent with the results of in vivo blood compatibility tests, no significant pathological abnormalities were observed in the H&E staining of main organs of KM mice (Figure 6d). The above in vitro and in vivo experimental data confirm that the synthesized composite microspheres have good biocompatibility, providing more evidence for the safe applications of PVA/MoS_2_ microspheres.

### 3.6. In Vitro Therapeutic Efficiency

**Figure 7:**
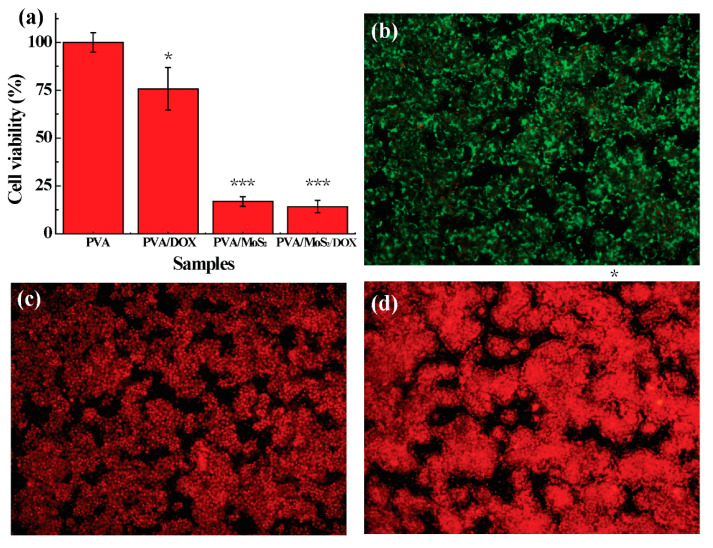
(**a**) The survival rate of HT 29 cells after treatments at noted. (**b**–**d**) Dead/Live staining of cells treated with (**b**) PVA/DOX, (**c**) MoS_2_ containing PVA microspheres, and (**d**) DOX and MoS_2_ containing PVA microspheres. The data were indicated with (*) for probability less than 0.05 (*p* < 0.05), and (***) for *p* < 0.001, respectively.

Prior to the application of electrospinning PVA/MoS_2_/DOX microspheres, we used HT29 cells and tumor-bearing nude mice to systematically study the therapeutic effects in vitro and in vivo. A strong red fluorescence was detected from the fluorescence photo of PVA/MoS_2_/DOX microspheres treated HT29 cells (Figure 7c,d). However, the red fluorescence signal of saline-treated HT29 cells was meaningless (Figure 7b). Therefore, the DOX released by PVA/MoS_2_/DOX microspheres can effectively kill the HT29 cells. After 24 h, the survival rate of HT29 cells treated with PVA/DOX decreased to 75.2 ± 6.4% (*p* < 0.05, versus control). After 5 min of exposure to the 808-nm laser, the survival rate of HT29 cells cultured with PVA/MoS_2_ microspheres was significantly reduced to 14.7 ± 1.3% (*p* < 0.01, versus control) due to the hyperthermia effect of the MoS_2_ nanosheets (Figure 7a). With the combination of the chemotherapy efficiency of DOX and the photothermal effect of MoS_2_ nanosheets, PVA/MoS_2_/DOX microspheres can further inhibit the proliferation and survival of colorectal cancer cells and the cell viability decreased to 13.4 ± 2.1% (*p* < 0.01, versus control). The Dead/Live staining results further prove the cell-killing effects of the PVA-based microspheres (Figure S3 and Figure 7b–d).

### 3.7. The Inhibition of Postoperative Tumor Recurrence

**Figure 8:**
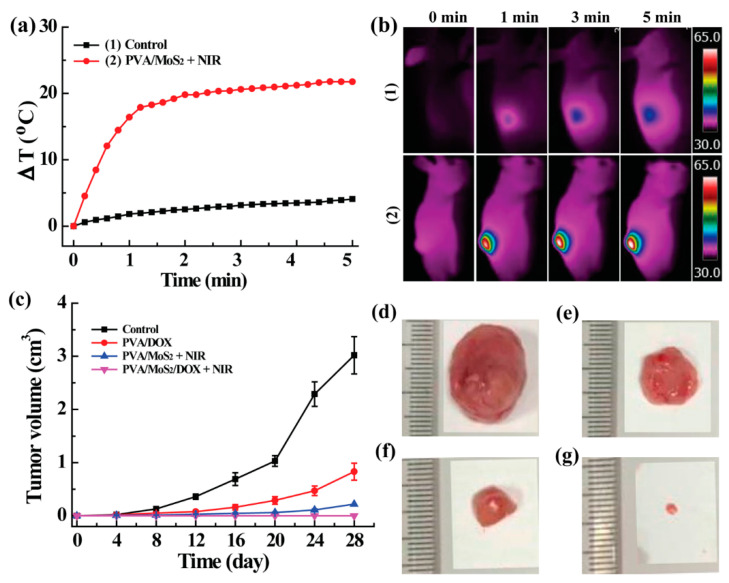
(**a**) The ΔT curves and (**b**) thermal pictures of untreated and MoS_2_-containing PVA-microspheres treated mice. (**c**) The real-time tumor volume of different treated mice. (**d**–**g**) The appearance of tumors at day 28 (treatments: (**d**), control; (**e**), PVA/DOX; (**f**), PVA/MoS_2_+ NIR; (**g**), PVA/MoS_2_/DOX + NIR).

We finally performed the in vivo experiments with tumor-bearing nude mice. Firstly, the in vivo photo-to-heat transformation of PVA/MoS_2_/DOX microspheres was studied. The results show that after the PVA/MoS_2_ microspheres were implanted in the tumor site, the stable photothermal conversion was well inherited in vivo. After the 30 s (1 W/cm^2^) laser irradiation, the temperature of the tumor site rapidly increased by 9.5 °C, and the maximum ΔT could reach 31.8 °C after 5 min. However, the maximum ΔT values of the control mice and mice treated with PVA/MoS_2_ microspheres were only 2.6 and 4.9 °C (Figure 8a). The infrared thermal imaging further confirmed that PVA/MoS_2_/DOX microspheres have an excellent in vivo photothermal conversion capability (Figure 8b). Due to the chemotherapy and photothermal effects of PVA/MoS_2_/DOX microspheres, mice implanted with PVA/MoS_2_/DOX microspheres showed no detectable tumor recurrence (Figure 8c,g), and the scars healed after being fed for 28 days. In contrast, tumors of untreated mice relapsed after 28 days of feeding, and the tumor volume expanded to 3.3 ± 0.4 cm^3^. Mice treated with PVA/DOX, PVA/MoS_2_ + NIR also showed the tumor recurrence to some extent and the volume expanded to 0.9 ± 0.2 cm^3^ and 0.3 ± 0.0 cm^3^, respectively (Figure 8c–g). Although in vivo experiments showed no significant difference between PVA/MoS_2_ + NIR and PVA/MoS_2_/DOX + NIR, the tumor therapy results indicate that PVA/MoS_2_/DOX nanosphere is a good candidate in the effective and safe inhibiting of the tumor recurrence in mice after colorectal surgery.

## 4. Conclusions

In general, a new kind of drug delivery composite, which is the PVA microsphere, was designed using the electrospinning and ball-mailing methods. The microspheres were further crosslinked with glutaraldehyde to obtain the water-stability. Uniform DOX and MoS_2_ co-loaded composite microspheres were easily prepared by doping MoS_2_ and DOX in the electrospinning solution. MoS_2_ nanosheets render the prepared PVA/MoS_2_/DOX microspheres an excellent light-to-heat conversion performance with η of 23.2%. In addition, the heat generated by NIR laser irradiation can improve the effect of chemotherapy by promoting the DOX release rate. HT29 cells and tumor-bearing nude mice were used to systematically study the combined tumor treatment efficiency of composite microspheres. The results showed that PVA/MoS_2_/DOX microspheres had excellent photothermal effect and chemotherapy effect, and can completely suppress the tumor recurrence. Because MoS_2_ and DOX were restricted to the tumor site by the polymer matrix, their damage to normal tissues was relieved. Therefore, the PVA/MoS_2_/DOX microspheres are anticipated to find potential applications in the treatment of local colorectal cancer.

## Data Availability

All data generated or analyzed during this study are included in this article.

## Supplementary Materials

The following are available online at https://www.mdpi.com/article/10.3390/pharmaceutics13070984/s1, Calculation of photothermal conversion efficiency (η). Figure S1: The routine blood parameters of healthy and treated mice. Treated mice are mice that were treated with PVA/MoS_2_/DOX microshperes. WBC: white blood cell; RBC: red blood cell; HCT: hematocrit; MCV: mean corpuscular volume; MCH: mean corpuscular hemoglobin; RDW: red cell distribution width. Figure S2: The routine blood parameters of healthy and treated mice. Treated mice are mice that were treated with PVA/MoS_2_/DOX microshperes. HB: hemoglobin; MCHC: mean corpuscular hemoglobin concentration; PLT: platelet. Figure S3: Dead/Live staining of cells in control group.
