# Supplementary material for: Novel PVA-Based Microspheres Co-Loaded with Photothermal Transforming Agent and Chemotherapeutic for Colorectal Cancer Treatment
Source: Pharmaceutics. 2021 Jun 29;13(7):984. doi: 10.3390/pharmaceutics13070984 (PMC8309159; doi:10.3390/pharmaceutics13070984)
Supplement: Supplementary file 1 [file pharmaceutics-13-00984-s001.zip › pharmaceutics-1233075-supplementary.pdf]

pharmaceutics

Supplementary Materials: Novel PVA-Based Microspheres Co-Loaded with Photothermal Transforming Agent and Chemo-therapeutic for Colorectal Cancer Treatment

Yao Zhang, Zirui He, Fan Yang, Changqing Ye, Xia Xu, Shige Wang, Ling Zhang and Duowu Zou

1. Calculation of photothermal conversion efficiency (η)

The η value of the microspheres was computed by a revised way similar to Korgel's report, where η can be calculated according to the following equation 1.

$$\eta = \frac{hS(T_{max}-T_{surr})-Q_{in,surr}}{I(1-10^{(-A(\lambda))})} \qquad (1)$$

In this equation, T_{max} in the unit of °C is the highest temperature that the microspheres reached during the laser irradiation and T_{surr} is the average temperature of ambient and $Q_{in,surr}$ is the heat lost to the surroundings when performing the experiment. I in the unit of mW stands for the 808 nm laser power and A(λ) represents the absorbance value of microspheres at the wavelength of 808 nm. S is the surface area of the microspheres and the value of hS was determined by measuring temperature-dropping rate upon switching off the 808 nm NIR laser. So hS can be calculated according to the following equation 2.

$$hS = \frac{m \cdot C_{H_2O}}{\tau_s} \qquad (2)$$

In this equation, m is the mass of the microspheres in units of g. C_{H2O} is the specific heat capacity of water in the unit of $J \cdot g^{-1} \cdot {}^{o}C^{-1}$. τs is the time constant of the system which can be calculated according to the following equation 3.

$$t = -\tau_s ln\theta = -\tau_s \ln\left(\frac{T-T_{surr}}{T_{max}-T_{surr}}\right) \qquad (3)$$

In this equation, t denotes the time in the cooling process (s). θ refers to the thermal drive constant. T is the real-time temperature of time t. Thus the value of τ_s was obtained by linearly fitting the negative value of the cooling time to the natural logarithm of the thermal drive constant.

2. Supplementary figures

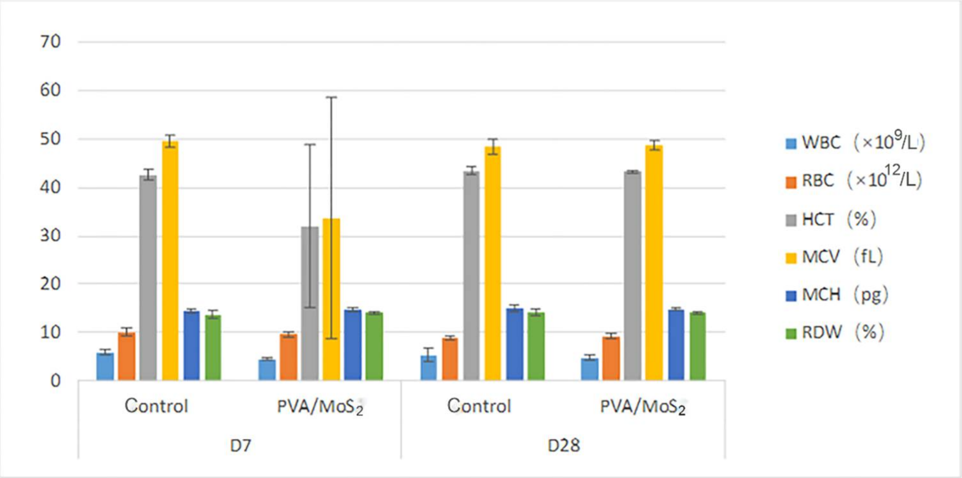

Figure S1. The routine blood parameters of healthy and treated mice. Treated mice are mice that were treated with PVA/MoS_2/DOX microshperes. WBC: white blood cell; RBC: red blood cell; HCT: hematocrit; MCV: mean corpuscular volume; MCH: mean corpuscular hemoglobin; RDW: red cell distribution width.

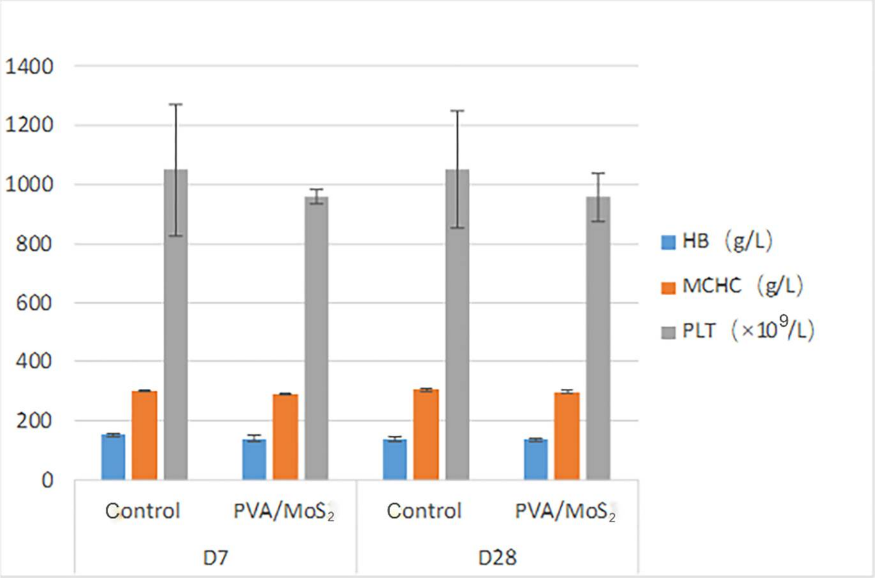

Figure S2. The routine blood parameters of healthy and treated mice. Treated mice are mice that were treated with PVA/MoS_2/DOX microshperes. HB: hemoglobin; MCHC: mean corpuscular hemoglobin concentration; PLT: platelet.

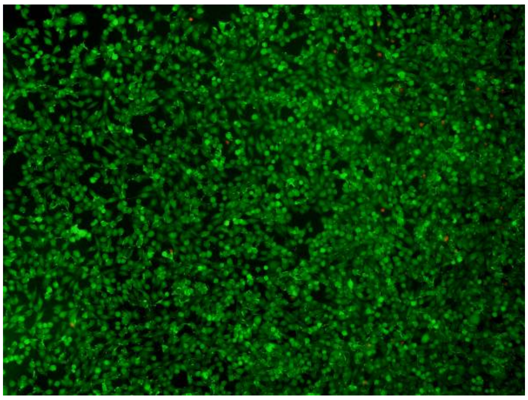

Figure S3. Dead/Live staining of cells in control group.
